# Business Co-Operation among Dentists—What the Sanitol Company Has Done for Dentist Stockholders

**Published:** 1907-07

**Authors:** 


### BUSINESS CO-OPERATION AMONG DENTISTS—WHAT THE SANITOL COMPANY HAS DONE FOR DENTIST STOCKHOLDERS.

The marvelous growth of the Sanitol Company has been remarked by the entire dental profession. Being a co-operative concern, having dentists as shareholders, the success of this business is particularly interesting to the dental profession.

Over five thousand dentists in this country have during the past ten years invested in the Sanitol Company. The foundation principles are profit-sharing and co-operation. The company has paid out to dentist stock-holders over $465,000 in dividends during the past ten years. No year has passed without a substantial dividend being paid. The Sanitol Company is the one successful co-operative company, and since its formation there have been hundreds of imitating concerns that have failed.

Dentists are peculiarly fitted to become interested in the Sanitol business, because the products manufactured by them are sold through the recommendations of the dental profession. An investment in Sanitol is not an experiment. It is a proven success. There is no industrial company in the world that has grown so rapidly during the past few years as has Sanitol.

Up to March 1, 1907, there was no opportunity open for new dentist stockholders. The entire capital stock of the company was subscribed. In March, 1907, however, the stockholders practically unanimously voted to increase the capital stock of the company.

The voted increase is now open for subscription.

The growth of the business has been so great during the past few years that the company was forced last year to begin the erection of a new plant. This plant has just been completed at a cost of $155,000 and with the new machinery and equippment, modern in every respect, added, it has brought the cost up to $200,000. In this new plant is a large room exclusively devoted to the use of dentists who are interested in the company. It comprises a suite of three large rooms, handsomely furnished, where dentists who are visiting St. Louis can spend their time, write their letters and make their headquarters. It will also be the stockholders' meeting room and can be obtained for conventions or local dentists' meetings.

